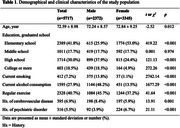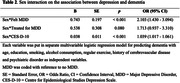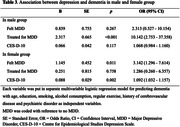# Sex differences in the association between depression and dementia

**DOI:** 10.1002/alz.095056

**Published:** 2025-01-09

**Authors:** Anna Jahng, Jaemyeong Kang

**Affiliations:** ^1^ University of California, Berkeley, Berkeley, CA USA; ^2^ UCSF, San Francisco, CA USA; ^3^ San Francisco Veterans Affairs Medical Center, San Francisco, CA USA; ^4^ Gil Medical Center, Gachon University College of Medicine, Incheon Korea, Republic of (South)

## Abstract

**Background:**

Sex differences in the association between depression and dementia have been reported with inconsistent results. This study aims to explore sex differences in the relationship between dementia and depression, using the history of major depressive disorder (MDD) and Centre for Epidemiological Studies Depression Scale (CES‐D‐10) as measures.

**Method:**

Utilizing data from the 8th wave of the Korean Longitudinal Study of Aging (KLoSA) conducted in 2020 with 5717 participants, we investigated the sex effect on the association between various measures of depression and history of dementia using binary logistic regression. Two depression history variables (‘Felt MDD’ and ‘Treated for MDD’ in the last two years) and one depressive symptom variable (CES‐D‐10) were used as independent variables. Subgroup analyses in each sex group were explored. All analyses were adjusted for age, education, current smoking and alcohol consumption, regular exercise, and history of cerebrovascular disease and psychiatric disorder. Additionally, Area Under the Receiver Operating Characteristic (AUROC) analyses were performed to investigate the predictive power of CES‐D‐10 for dementia.

**Result:**

No significant differences were observed between males and females for the prevalence of ‘Felt MDD,’ ‘Treated for MDD,’ CES‐D‐10 score, and history of dementia. While significant sex (female) interaction was detected in the association between ‘Felt MDD’ and dementia (Odds Ratio [OR] 2.103, 95% Confidence Interval [CI] 1.430 – 3.094) and between CES‐D‐10 and dementia (OR 1.039, CI 1.017 – 1.061), no significant sex interaction was found in the association between ‘Treated for MDD’ and dementia (OR 1.713, CI 0.937 – 3.310). Subgroup analyses revealed a significant association between ‘Treated for MDD’ and dementia (OR 10.142, CI 2.753 – 37.358) in males. In females, ‘Felt MDD’ (OR 3.142, CI 1.296 – 7.614) and CES‐D‐10 (OR 1.092, CI 1.032 – 1.157) showed significant association with dementia. Also, CES‐D‐10 demonstrated higher AUC predicting dementia in females (0.705) compared to males (0.666) or both sexes combined (0.692).

**Conclusion:**

A greater association was found between depression and dementia in the female group compared to the male group. This might imply that sex plays a role in the association between depression and dementia.